# Grading of aortic regurgitation by cardiovascular magnetic resonance and pulsed Doppler of the left subclavian artery: harmonizing grading scales between imaging modalities

**DOI:** 10.1007/s10554-020-01844-2

**Published:** 2020-04-18

**Authors:** Ricardo A. Spampinato, Cosima Jahnke, Ingo Paetsch, Sebastian Hilbert, Susanne Löbe, Frank Lindemann, Elfriede Strotdrees, Gerhard Hindricks, Michael A. Borger

**Affiliations:** 1 Department of Cardiac Surgery, University of Leipzig – HELIOS Heart Center, Strümpellstraße 39, 04289 Leipzig, Germany; 2 Department of Cardiology/Rhythmology, University Leipzig – HELIOS Heart Center, Leipzig, Germany

**Keywords:** Aortic valve regurgitation, Left subclavian artery, Quantification, Doppler, Echocardiography, CMR

## Abstract

Transthoracic echocardiography (TTE) and cardiac magnetic resonance (CMR) are current standard for assessing aortic regurgitation (AR). Regurgitant fraction (RF) can also be estimated by Doppler examination of the left subclavian artery (LSA-Doppler). However, a comparison of AR grading scales using these methods and a TTE multiparametric approach as reference is lacking. We evaluated the severity of AR in 73 patients (58 ± 15 years; 57 men), with a wide spectrum of AR of the native valve. Using a recommended TTE multiparametric approach the AR was divided in none/trace (n = 12), mild (n = 23), moderate (n = 12), and severe (n = 26). RF was evaluated by LSA-Doppler (ratio between diastolic and systolic velocity–time integrals) and by CMR phase-contrast imaging (performed in the aorta 1 cm above the aortic valve); the grading scales were then calculated. There were a good correlation between all methods, but mean RF values were greater with TTE compared with LSA-Doppler and CMR (39 ± 16% vs. 35 ± 18% vs. 32 ± 20%, respectively; p < 0.037). Mean differences in RF values between methods were significant in the groups with mild and moderate AR. Grading scales that best defined the TTE derived AR severity using CMR were: mild, < 21%; moderate, 22 to 41%; and severe, > 42%; and using LSA-Doppler: mild, < 29%; moderate, 30 to 44%; and severe, > 45%. RF values for AR grading using TTE, LSA-Doppler and CMR correlate well but differ in groups with mild and moderate AR when using a recognized multiparametric echocardiographic approach. Clinical prospective studies should validate these proposed modality adjusted grading scales.

**Electronic supplementary material:**

The online version of this article (10.1007/s10554-020-01844-2) contains supplementary material, which is available to authorized users.

## Introduction

Transthoracic echocardiography (TTE) is widely recognized as non-invasive gold standard for quantification of aortic valve regurgitation (AR). The severity of AR can also be evaluated with the estimation of regurgitant fraction (RF) by flow measurement in ascending aorta using cardiovascular magnetic resonance imaging (CMR). Recently, was also validated the assessment of the RF by examination of the left subclavian artery (LSA) velocity contour by pulsed Doppler as an alternative method [1, 2].

However, there is still no final consensus in which scale should be used with these modalities to define AR severity. Several studies compared AR severity by CMR vs. angiography and/or echocardiography. But the cut-off values reported are variable [3], reflecting the variability of the reference parameters to which CMR was compared. It ranged from pure qualitative through semi-quantitative to pure quantitative echocardiographic parameters. As a result, the cut-off value for severe AR on CMR varies from 30 to 50% [4–8]. Thereby, the writing groups for the evaluation of native valvular regurgitation and after percutaneous valve repair or replacement [9, 10] had to reach consensus to use the same partition for AR severity as recommended for echocardiography until further data are available.

All proposed echocardiographic qualitative, semi-quantitative, and quantitative methods exhibit significant limitations. They are not feasible in a percentage of patients with AR, principally, due to inappropriate acoustic windows, interposition of valve tissue, and the inherent difficulty in correctly identifying the flow convergence zone. Hence, current echocardiographic guidelines strongly recommend an *integrative* approach using multiple qualitative, semi-quantitative, and quantitative measurements [10, 11].

A comparison of the RF measurements of above mentioned methods with a recommended TTE *multiparametric* approach has not been evaluated. Consequently, we sought to analyze which grading scale of CMR and LSA-Doppler derived RF best correlates with mild, moderate, and severe grades of AR, using a *multiparametric* echocardiographic approach as a reference standard.

## Material and methods

### Study population

We prospectively enrolled 73 patients (58 ± 15 years; 57 men), 61 with a wide spectrum of AR of the native valve referred to our center for evaluation of the pathology (AR group), and 12 patients with non or trace AR on TTE with clinical indication for a CMR study other than heart valve disease (control group). Patients were eligible if they had sinus rhythm during the study. Those with an associated cardiac valve lesion more than moderate, with aortic coarctation, or with typical contraindications for CMR imaging were excluded.

Usually, patients underwent CMR imaging, LSA-Doppler, and TTE within 12 h. All baseline characteristics were prospectively collected. Based on a recommended TTE *multiparametric* approach the AR was quantified and divided in to 3 groups: mild (n = 23), moderate (n = 12), and severe (n = 26). We evaluated also the RF derived from the Doppler examination of the LSA (ratio between diastolic and systolic velocity–time integrals) and from CMR phase-contrast quantitative flow (performed in the aorta 1 cm above the aortic valve). RF measurements of all methods were then compared within the groups.

### Pulsed Doppler of the left subclavian artery velocity contour

The systolic and diastolic flow profiles of the LSA were evaluated by use of pulsed wave Doppler ultrasound (3.4–9 MHz linear probe). Patients were examined in a supine position with a subclavicular approach. Higher-frequencies (> 7 MHz) were used for assessment of the morphology, and lower-frequency (< 7 MHz) was preferred for Doppler examination. LSA was documented with gray-scale imaging and color Doppler to rule out relevant stenosis. The depiction of the LSA was modified to align the Doppler angle parallel to the vector of blood flow and to avoid the Doppler signal of the adjacent vein. The sample volume was placed just nearby the origin of the LSA. Patients with vascular shunts of the left upper arm were excluded. As previously described [2], the outer edge of the dense envelope of the spectral recording (i.e. modal velocity) was used to measure the VTI [12]. The RF was calculated as follows: RF(%) = LSA derived diastolic reversed flow VTI × 100/LSA systolic forward flow VTI. At least two measurements were performed. The LSA-Doppler examination was carried out blindly to the results of echocardiography and the CMR evaluation of AR.

### Standard echocardiography

All TTE were performed by experienced echocardiographers using commercially available ultrasound machines (Vivid E9, General Electric Healthcare, Wauwatosa, USA; or Acuson SC2000, Siemens Healthcare GmbH Erlangen, Germany) equipped with M5S or 4V1c 2D TTE probes. All recordings were stored digitally for offline analysis. Left-ventricular (LV) volumes and ejection fraction (EF) were calculated using the biplane Simpson disk method. Doppler measurements were evaluated as the average of at least three cycles. An effort was made to perform the flow convergence (PISA) method, from apical views or, in case of eccentric jets, from parasternal long-axis views. The AR severity was graded according to a recommended TTE “multiparametric approach” [10, 11], conceivable as comprehensive and integrative process, based on structural (i.e. aortic valve morphology and LV size), qualitative (i.e. Jet width and density, Jet deceleration rate -PHT-), semiquantitative (i.e. vena contracta width, end-diastolic flow velocity in the descending aorta), quantitative parameters (flow convergence method and quantitative pulsed Doppler), and all adjunctive parameters collected during the echocardiographic examination to consolidate the evaluation of the severity of AR. In intermediate cases the quantitative methods were conclusive. Evaluation of AR was performed by an experienced echocardiographer (RS, > 10 years of experience in echocardiography with ESC certification), who was blinded to the results of the CMR and LSA Doppler exams.

### Cardiovascular MR

All CMR examinations were performed in our cardiology department on a 1.5-T MRI system (Ingenia, Philips Healthcare, Best, the Netherlands) equipped with a 28-element array coil with full in-coil signal digitalization combined with optical transmission. All scans were accomplished without sedation. Image data acquisition and subsequent analysis were carried out according to current guidelines. For cine imaging, a balanced steady-state free precession (b-SSFP) sequence with retrospective gating was used during short-periods of breath-holding. All standard cardiac geometries were acquired (multiple, gapless short-axis slices covering the entire left ventricle and 2-, 3- and 4-chamber views). Imaging parameters were chosen as follows: echo time (TE) and repetition time (TR) were set to shortest resulting in an average TR of around 4 ms and a TE of 2 ms, with a reconstructed in-plane resolution of 1.0 × 1.0 mm^2^; the slice thickness was 8.0 mm. The typical temporal resolution of the cine b-SSFP sequences was 30–25 ms depending on the heart rate. The imaging plane for the through-plane phase-contrast flow measurement was placed in the ascending aorta approximately 10 mm above the aortic valve and positioned perpendicular to the flow direction. On a coronal image of the aorta together with the 3-chamber view the CMR operator checked that the image plane was truly perpendicular to the aortic flow direction. To avoid aliasing, velocity encoding was individually adapted, starting at 200 cm/s, and if aliasing occurred, the maximum velocity was increased by 50 cm/s steps until aliasing disappeared. Image data acquisition was gated to the ECG signal with a temporal resolution of 35 phases per cardiac cycle and acquired during a 12–15 s breath-hold. Outlining the region of interest within the aortic lumen for each cardiac phase, the instantaneous flow volume (cm^3^/s) was calculated and graphically displayed over the entire cardiac cycle.

Forward and reversed flow volumes were measured, and the regurgitation fraction was calculated as follows: aortic RF (%) = diastolic reversed flow volume × 100/systolic forward flow volume.

### Statistical analysis

Data are presented as mean (SD), median (25th to 75th percentile), or frequency (percent) as appropriate. Statistical differences between groups were assessed using Student’s t-test for continuous variables or Fisher’s exact test for categorical variables. Multigroup comparisons of continuous variables were performed using an analysis of variance (ANOVA). Spearman correlation coefficient was used to assess correlations between methods. Linear regression and c-statistics were used to assess the cut-off values of CMR and LSA-Doppler derived RF that best reflect the three-scale grading of AR by multiparametric TTE approach. Using these cut-offs the rate of agreement for AR grading was evaluated by calculating a κ-statistics. Additionally, the mean difference with the 95% confidence interval was also determined. Two-tailed p-values < 0.05 were considered statistically significant. Analyses were performed using SPSS software (IBM-SPSS Statistics, Version 20, IBM Corp.). The study was conducted in accordance with the Declaration of Helsinki, and was approved by the local research ethics committee (067/17-ek). All patients received informed consent.

## Results

**Fig. 1:**
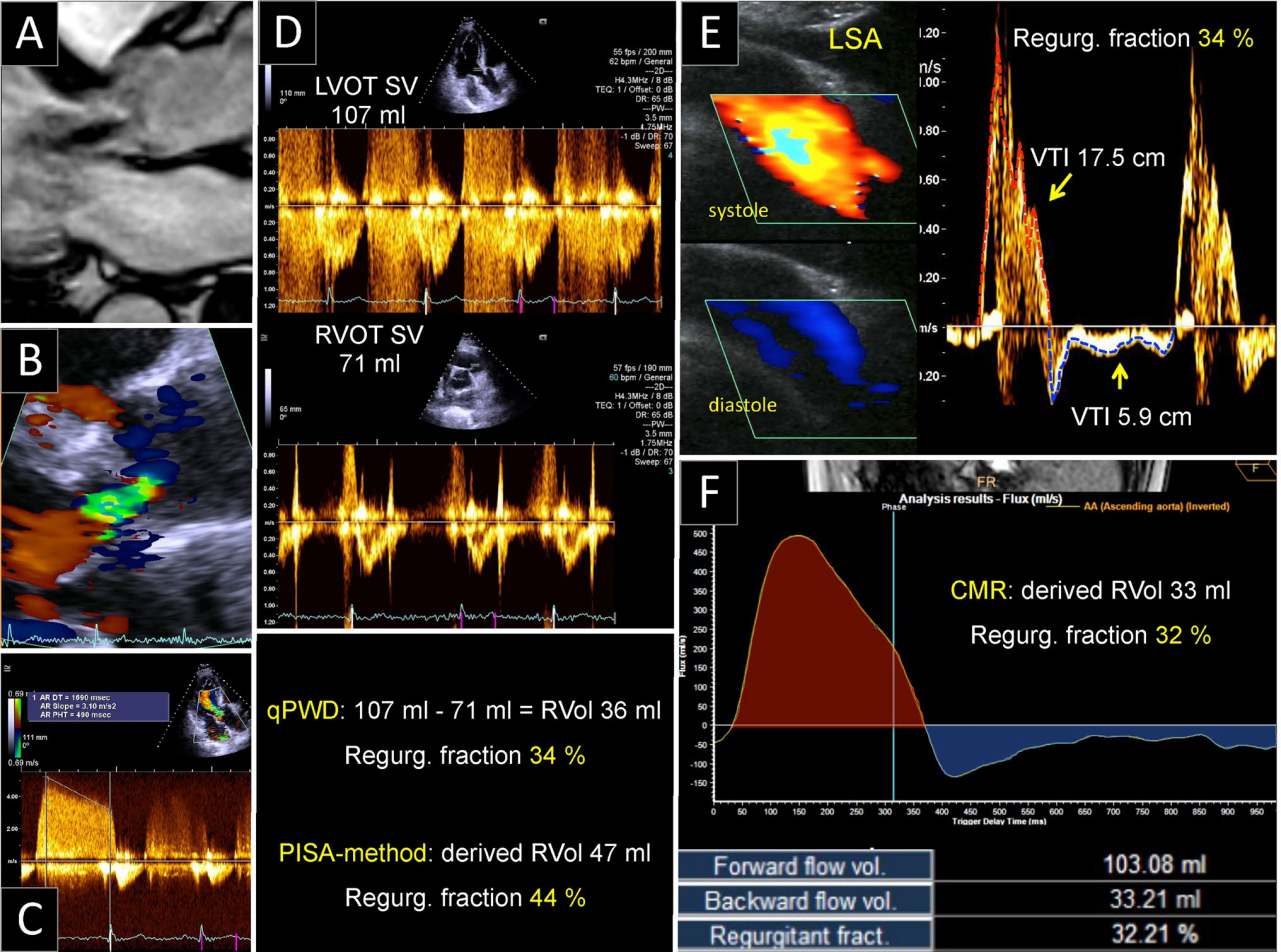
Example of a case with moderate aortic regurgitation (AR). **A**, **B** AR-jet in CMR and TTE 3-chamber views. **C** Continuous-wave Doppler velocity contour of the regurgitant jet depicting a pressure half time of approx. 500 ms. **D** Quantitative pulse-wave Doppler (qPWD) at the level of the left ventricle (LVOT) and right ventricle outflow tract (RVOT). **E** Left subclavian artery (LSA) Doppler method. Color Doppler and LSA velocity contours in systole (red) and diastole (blue) are depicted. The correspondence forward and backward VTI are traced from modal velocity. **F** CMR Q-flow derived regurgitant fraction. *CMR* cardiovascular magnetic resonance, *TTE* transthoracic echocardiography, *VTI* velocity time integral, *RVol* regurgitant volume, *SV* stroke volume

**Fig. 2:**
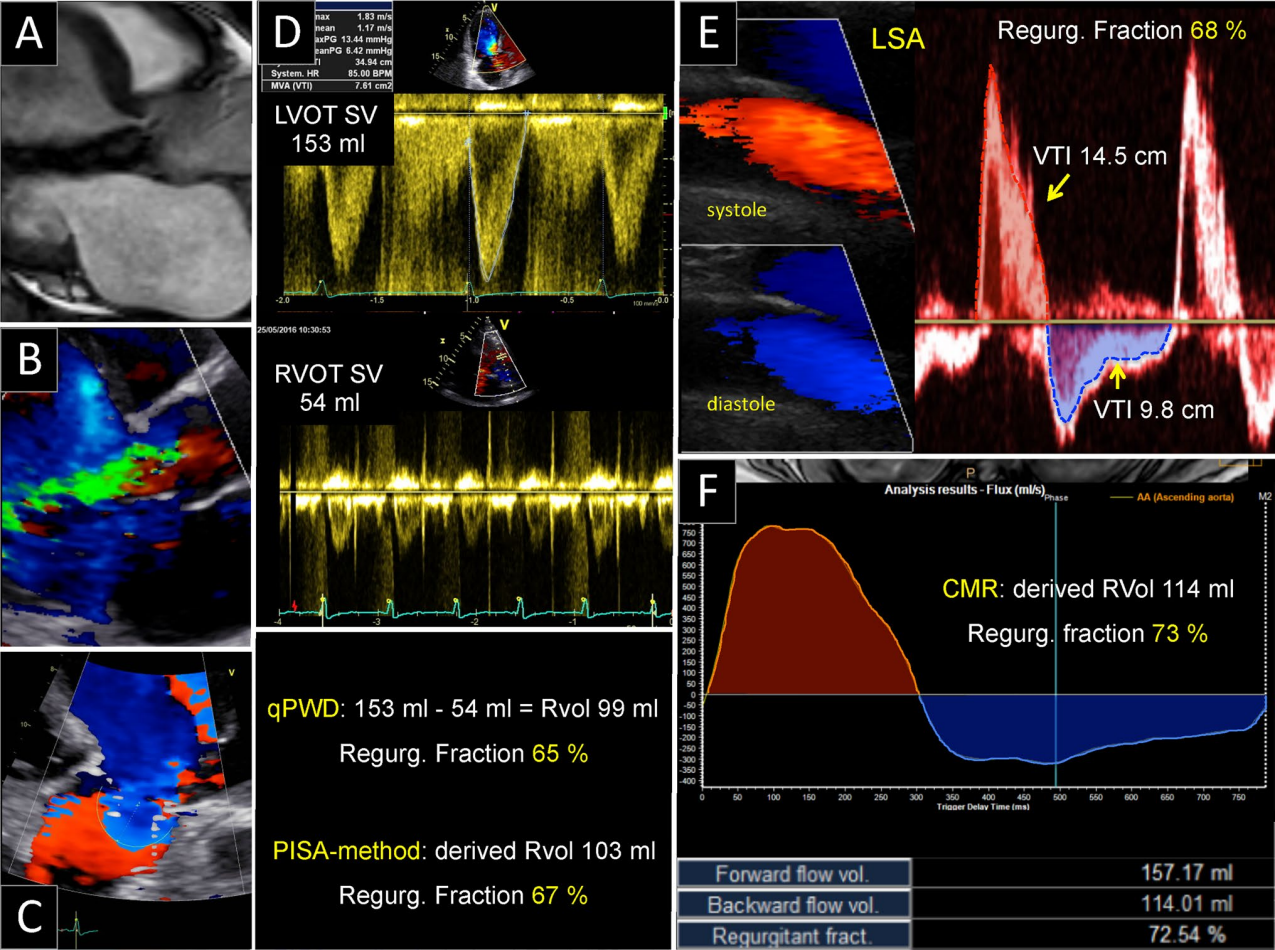
Example of a case with severe aortic regurgitation (AR). **C** Color Doppler depicting the flow convergence region of the AR from an apical log axis. Other references and abbreviations as in Fig. 1

Demographic and baseline patient characteristics are presented in Table 1. There were no significant differences between controls and AR group, with exception of a higher rate of hypertension in the AR group. A high quality CMR study and Doppler signal of the LSA could be obtained in all patients. PISA method could be obtained in 85% of AR group´s participants. Echocardiographic characteristics of all groups are shown in Table 2. Patients with severe AR had higher TTE and CMR derived LV end-diastolic and stroke volume, without differences in LV–EF. Figures 1 and 2 show two examples of cases with moderate and severe AR.

**Table 1:** Patient characteristics

	Controls (n = 12)	AR group (n = 61)
Age, years	51 ± 17	59 ± 14
Male, n (%)	7 (58)	50 (82)
CAD, n (%)	0	12 (20)
Hypertension, n (%)	4 (33)	49 (80)*
Diabetes, n (%)	0	7 (11.5)
Dyslipidemia, n (%)	2 (17)	26 (43)
BSA, m2	1.91 ± 0.2	1.99 ± 0.2
Aorta diameter, mm	31 ± 4	37 ± 7
Aortic valve morphology		
Tricuspid, n (%)	12 (100)	42 (72)
Bicuspid, n (%)	0	15 (26)
Quadricuspid, n (%)	0	1 (1.7)
Undefined, n (%)	0	3 (6.4)

Unless otherwise specified, values are expressed as mean ± SD

*AR* aortic regurgitation, *CAD* coronary artery disease, *BSA* body surface area

*p < 0.05

**Table 2:** Multiparametric TTE classification of AR

	Control (12)	AR group (61)	Mild (23)	Moderate (12)	Severe (26)	p value^a^
Echocardiographic parameters						
EDV, ml	116 ± 39	196 ± 81	137 ± 34	169 ± 49	260 ± 76	< 0.0001
ESV, ml	53 ± 31	88 ± 48	61 ± 22	73 ± 26	119 ± 54	< 0.0001
LVSD, mm	36 ± 7	42 ± 9	37 ± 6	38 ± 8	49 ± 6	< 0.0001
EF, %	56 ± 14	56 ± 10	56 ± 8	56 ± 9	56 ± 11	0.997
Stroke Volume, ml	70 ± 20	120 ± 44	87 ± 21	108 ± 28	156 ± 38	< 0.0001
EROA, cm^2^*	n.a	0.27 ± 0.18	0.08 ± 0.04	0.24 ± 0.07	0.43 ± 0.14	< 0.0001
RV, ml*	n.a	52 ± 33	17 ± 8	43 ± 10	82 ± 21	< 0.0001
RF, %*	n.a	**39 ± 16**	**19 ± 6**	**39 ± 6**	**54 ± 7**	< 0.0001
Vena contracta, mm	n.a	4.3 ± 1.8	2.6 ± 0.6	3.7 ± 0.9	6.1 ± 0.9	< 0.0001
PHT, ms	n.a	436 ± 196	642 ± 153	407 ± 94	285 ± 87	< 0.0001
QP/QS	1.02 ± 0.06	0.65 ± 0.2	0.86 ± 0.08	0.66 ± 0.06	0.45 ± 0.07	< 0.0001
CMR parameters						
EDV, ml	161 ± 39	244 ± 101	170 ± 36	196 ± 52	331 ± 91	< 0.0001
ESV, ml	78 ± 42	119 ± 66	77 ± 22	90 ± 28	168 ± 71	< 0.0001
EF, %	54 ± 11	53 ± 9	55 ± 8	54 ± 8	51 ± 11	0.455
RF, %	**3 ± 2**	**32 ± 20**	**11 ± 7**	**30 ± 10**	**50 ± 9**	< 0.0001
Aorta forward flow, ml	83 ± 22	122 ± 48	92 ± 20	98 ± 28	159 ± 47	< 0.0001
Aorta backward flow, ml	2 ± 1.8	44 ± 38	10 ± 7	30 ± 14	81 ± 29	< 0.0001
LSA Doppler						
Forward VTI, cm	16.4 ± 3	20.4 ± 7	21 ± 8	19 ± 6	21 ± 6	0.178
Backward VTI, cm	1.3 ± 0.6	7 ± 4	3.3 ± 2	6.4 ± 2	11 ± 3	< 0.0001
RF, %	**7.6 ± 3**	**35 ± 18**	**17 ± 8**	**35 ± 6**	**51 ± 10**	< 0.0001

Bold values indicate the RF values by all methods

Echocardiographic, CMR, and LSA Doppler values

Values are expressed as mean ± SD

*TTE* transthoracic echocardiography, *AR* aortic regurgitation, *CMR* cardiac magnetic resonance, *LSA* left subclavian artery, *EF* left ventricular ejection fraction, *EDV* LV end-diastolic volume, *ESV* LV end-systolic volume, *LVSD* LV end-systolic diameter, *EROA* effective regurgitant orifice area, *RV* regurgitant volume (AR), *RF* regurgitant fraction (AR), *QP/QS* pulmonary flow (QP)/systemic flow (QS) ratio, *PA* pulmonary artery, *VTI* velocity time integral

*Calculated with PISA method (n = 52)

^a^Within AR groups

### Agreement between methods

**Fig. 3:**
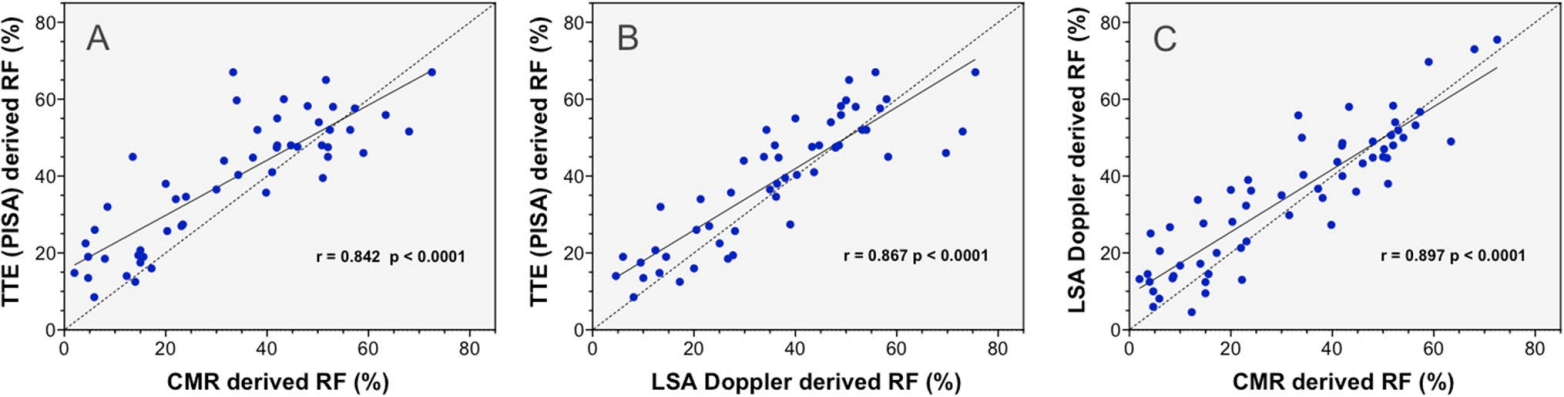
Correlation for aortic regurgitant fraction (RF) measurements variability with echocardiography (TTE) derived convergence method (PISA), cardiac magnetic resonance (CMR), and left subclavian artery Doppler (LSA-Doppler). Dashed line indicates line of identity; and solid line, linear regression line. Pearson correlation (r) between PISA and CMR (**A**), PISA and LSA-Doppler (**B**), and LSA-Doppler and CMR (**C**) are showed

**Fig. 4:**
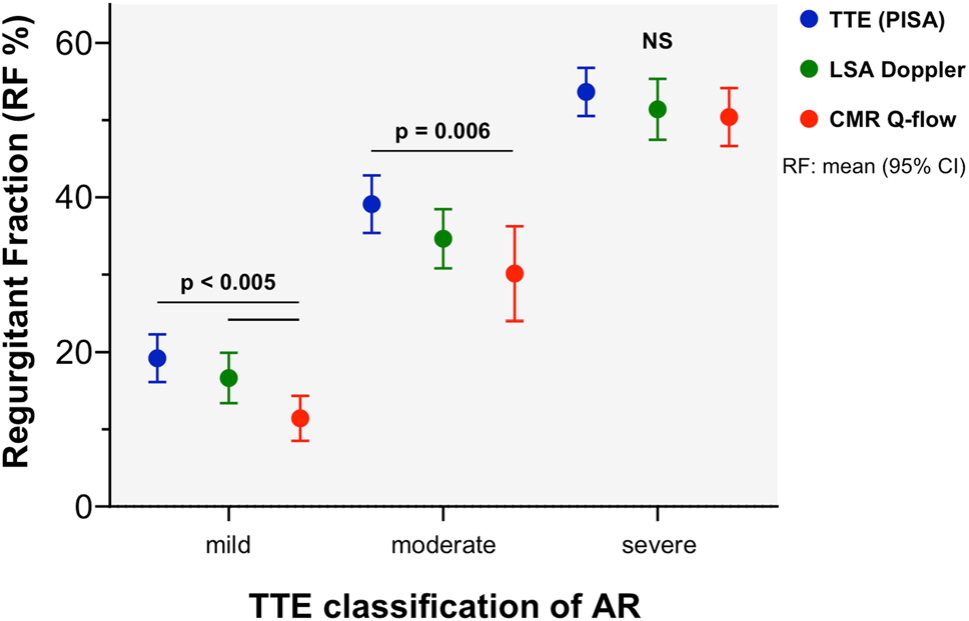
Comparison of aortic regurgitant fraction (RF) as determined by echocardiographic (TTE) flow convergence method (PISA, in blue), left subclavian artery Doppler (LSA-Doppler, in green), and cardiac magnetic resonance quantitative flow (CMR Q-flow, in red), according to the aortic regurgitation (AR) grade as determined by multiparametric TTE approach. Errors bars represent RF mean values and 95% CI. NS: not significant

In the whole cohort the RF measurements assessed by PISA-method, LSA-Doppler, and CMR imaging were strongly correlated (Fig. 3). But the mean RF values were significantly greater on TTE compared with LSA-Doppler and CMR (39 ± 16% vs. 35 ± 18% vs. 32 ± 20%, respectively; p < 0.037). The mean differences of RF values were significant in the groups with mild and moderate AR, with overestimation by TTE (Table 3 and Fig. 4).

**Table 3:** Aortic regurgitant fraction (RF) measurements using PISA method, LSA Doppler, and CMR stratified by severity grade using a multiparametric transthoracic echocardiographic approach

	Mild AR		Moderate AR		Severe AR	
	Mean difference	p value	Mean difference	p value	Mean difference	p value
PISA vs. LSA Doppler	2.7 (− 1.1 to 6.6)	0.146	4.5 (− 0.5 to 10)	0.073	1.6 (− 3 to 6.2)	0.465
PISA vs. CMR	8.0 (4.1 to 12)	**< 0.001**	9.0 (3.1 to 15)	**0.006**	3.3 (− 2 to 9)	0.212
LSA Doppler vs. CMR	5.2 (1.9 to 8.6)	**0.004**	4.5 (− 2.0 to 11)	0.153	1.1 (− 2.4 to 4.4)	0.547

Bold values indicate all p values < 0.05

Data are presented as mean (95% confidence interval)

*AR* aortic regurgitation, *PISA* proximal isovelocity surface area, *CMR* cardiac magnetic resonance, *LSA* left subclavian artery

**Fig. 5:**
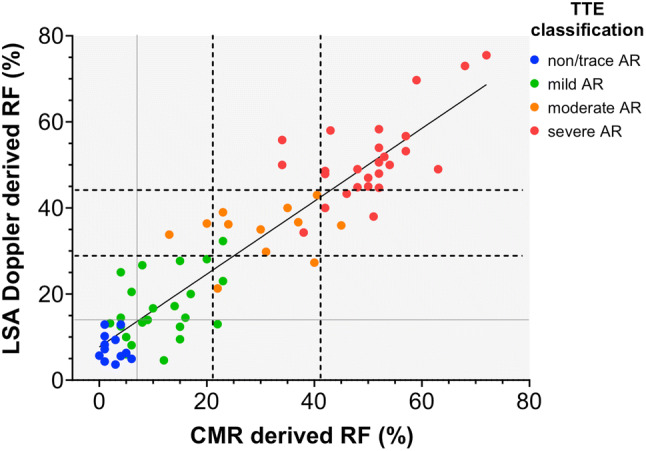
Linear regression of measurements of aortic regurgitant fraction (RF) by left subclavian artery Doppler (LSA) and cardiovascular magnetic resonance (CMR) imaging, according to multiparametric echocardiography (TTE) derived aortic regurgitation (AR) classification (none/trace AR, blue dots; mild AR, green dots; moderate AR, orange dots; and severe AR, red dots)

Using a linear regression and a receiver operating characteristic (ROC) curve analysis the RF values that best defined the multiparametric TTE derived AR severity using CMR were: mild, < 21%; moderate, 22 to 41%; and severe, > 42%; and using LSA-Doppler: mild, < 29%; moderate, 30 to 44%; and severe, > 45% (Fig. 5). Applying these RF cut-offs, the level of agreement between LSA-Doppler, CMR, and multiparametric TTE grading of AR was strong (Table 4). Importantly, in patients with severe AR, there were only three misclassified patients by LSA-Doppler and CMR, resulting in a good sensitivity, specificity, and diagnostic accuracy for severe AR (88.5%, 94.3%, and 91.8%; respectively). The ROC curve analysis showed similar cut-off values for severe AR when using the best sum of sensitivity and specificity giving an excellent area under the curve. Applying the same grading scale for all methods (mild, < 30%; moderate, 31 to 49%; and severe, > 50%) the level of agreement between modalities was numerically lower with kappa values of 0.628 and 0.616 for CMR and LSA Doppler, respectively (Online Resource 1).

**Table 4:** Grading of aortic regurgitation severity: Agreement of a multiparametric transthoracic echocardiography approach (TTE) with cardiovascular magnetic resonance (CMR), Doppler of the left subclavian artery (LSA), and with flow convergence (PISA) method

	TTE				S	M–S
	Mild AR	Moderate AR	Severe AR			
CMR^a^						
Mild	**19**	1		20 (32.8%)	Sensitivity 88.5%	97.4%
Moderate	4	**9**	3	16 (26.2%)	Specificity 94.3%	82.6%
Severe		2	**23**	25 (41.0%)	Accuracy 91.8%	91.8%
	23 (37.7%)	12 (19.7%)	26 (42.6%)	**51/**61 (**83.6%**)		
LSA Doppler^b^						
Mild	**17**			17 (27.9%)	Sensitivity 88.5%	100%
Moderate	6	**10**	3	19 (31.1%)	Specificity 94.3%	73.9%
Severe		2	**23**	25 (41.0%)	Accuracy 91.8%	90.2%
	23 (37.7%)	12 (19.7%)	26 (42.6%)	**50/**61 (**82.0%**)		
PISA method^c^						
Mild	**15**			15 (28.8%)	Sensitivity 69.6%	100%
Moderate	2	**11**	7	20 (38.5%)	Specificity 96.6%	88.2%
Severe		1	**16**	17 (32.7%)	Accuracy 84.6%	96.2%
	17 (32.7%)	12 (23.1%)	23 (44.2%)	**42/**52 (**80.8%**)		

Bold values indicate all cases with concordance between methods

Aortic regurgitant fraction cut-off: *CMR* mild, < 21%; moderate, 22 to 41%; and severe, > 42%, *LSA-Doppler* mild, < 29%; moderate, 30 to 44%; and severe, > 45%, *PISA-method* mild, < 30%; moderate, 31 to 49%; and severe, > 50%

Diagnostic test analyses: first column *S*, for severe AR, second column *M–S*, for moderate to severe AR

^a^Kappa 0.748 (p < 0.0001); feasibility 100%

^b^Kappa 0.726 (p < 0.0001); feasibility 100%

^c^Kappa 0.714 (p < 0.0001); feasibility 85.2%

Seven of ten patients, with PISA derived RF just below the recommended limit for severe AR (borderline values of 45 to 48%), were recategorized as severe AR when a multiparametric approach was used. Five of these 7 patients were symptomatic; all of them had dilated LV (LV end-diastolic volume 295 ± 55 ml, LV end-systolic diameter 48 ± 6 mm); and the decision to grade them as severe AR were made based on proposed echocardiographic parameters like a descending aorta end-diastolic flow reversal velocity > 20 cm/s, quantitative Doppler derived RF > 50%, and a vena contracta > 6 mm. Finally, there were comparable feasibility and kappa values for the agreement between all three methods and multiparametric TTE approach (Table 4).

On the other extreme of the grading scale there was a significant overlap between non/trace AR (control group) and mild AR. RF cut-offs of < 7% for CMR and < 14% for LSA-Doppler showed a sensitivity and a positive predictive value of 100% for presence of non/trace AR but a poor specificity (59% for CMR, 47% for LSA-Doppler) due to overlap between non/trace and mild AR (Fig. 5).

## Discussion

To the best of our knowledge, this is the first study to analyze which grading scale best defines the *multiparametric* TTE derived AR severity using CMR imaging and the recently proposed LSA-Doppler method in adult patients with a wide spectrum of native aortic valve regurgitation. The proposed scale for CMR were: mild, ≤ 21%; moderate, 22 to 41%; and severe, ≥ 42%; and for LSA-Doppler: mild, ≤ 29%; moderate, 30 to 44%; and severe, ≥ 45%. Using these grading scales and a multiparametric TTE approach as the standard of reference, AR was graded correctly in 51 of 61 patients (83.6%) applying CMR imaging, and in 50 of 61 patients (82%) with LSA-Doppler method. The kappa values of 0.748 and 0.726 indicate good agreement. Harmonizing grading schemes between imaging modalities. Indeed, using a unified standard grading scale for all methods as proposed in current recommendations [9, 10] resulted in a lower level of agreement between CMR, LSA Doppler, and multiparametric TTE. A cut-off for severe AR set to a RF > 50% led to a lower sensitivity of up to 60% and 50%, by CMR and LSA Doppler respectively (Online Resource 1). Meaning that the use of the same cut-off by CMR imaging for the detection of severe AR, could lead to an underestimation of severe AR in approximately 40% of patients with significant AR, which may lead to a late referral to surgery.

**Fig. 6:**
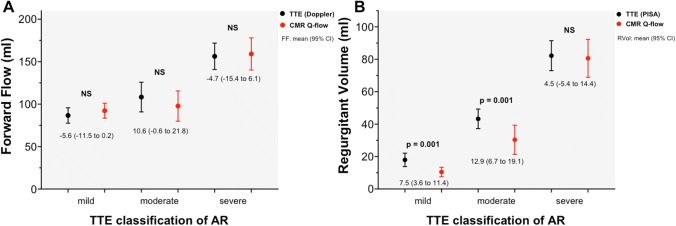
Comparison of aortic forward flow (FF, left) and regurgitant volume (RVol, right) as determined by echocardiographic (TTE) Doppler or flow convergence (PISA) method (in black) and cardiac magnetic resonance (CMR, in red), according to the aortic regurgitation (AR) severity as determined by multiparametric TTE approach. Errors bars represent mean values and 95% CI. Mean differences (95% CI) are expressed under the errors bars. NS: not significant

The feasibility of the PISA method was 85%, in accordance with original publications [13]. But a significant overestimation of the PISA derived RF in the group with mild and moderate AR was observed. There is some evidence that PISA method might overestimate the regurgitant volume and fraction when comparing with CMR, as has been noted for primary mitral regurgitation [14]. The overestimation of RF with PISA could be secondary to underestimation of the LV outflow tract (LVOT) Doppler derived stroke volume (SV) or overestimation of the PISA derived RVol. In our study there were no differences between Doppler derived LVOT SV and CMR aortic forward flow, and higher values of the PISA derived RVol were observed (Fig. 6), which reflects an intrinsic association with the flow convergence method.

LSA-Doppler method also displayed higher RF values in the group with mild AR. The more pronounced the vessel wall elasticity the more expansion in systole and recoil in diastole. This means that the diastolic VTI is recorded from a smaller area than the systolic VTI, implying that the diastolic/systolic VTI ratio should overestimate the RF. A simplified formula has been proposed for correcting for aortic pulsatility [15] but specially in younger patients, without history of hypertension, and with moderate to severe AR.

Discrepancies in the group of mild and moderate AR might also be explained with the fact that CMR phase-contrast sequences may underestimate the slower, swirling regurgitant flow. Moreover, blood ejected into the aortic sinuses in systole that has not yet crossed the CMR image slice flows back into the left ventricle and, hence, will not contribute to the AR determination: the smaller RVol and RF, the greater the influence on the diagnostic accuracy. These potential limitations may be overcome by the use of slice tracking flow CMR sequences, which follow the valve and capture this potentially “undetected” regurgitation, with an increase in the RF by 60%, 15%, and 7% in mild, moderate and severe AR, respectively [16].

Interestingly, in the group with severe AR there were no significant differences between all three methods derived RF. Suggesting that the cut-off value for severe AR should be relative similar with all methods. Indeed, by PISA method the cut-off value for severe AR with the best sum of sensitivity and specificity, using a multiparametric TTE approach as reference, was a RF > 46%. This cut-off value slightly lower than the recommended by the guidelines was due to the presence of seven patients with borderline PISA derived RF values that were recategorized as severe AR according to the multiparametric TTE approach. Indeed, a trend toward underestimation of the RVol by PISA was observed [13] in patients with shadowing by the aortic wall, especially in case of bigger flow convergence region by severe AR with wall impingement. Eccentric wall-hugging jets lose momentum rapidly, thus appear smaller than non-constrained jets with same RVol. Another important consideration is the angle of interrogation: since color Doppler is sensitive only to the component of flow in the direction of the transducer, jets interrogated orthogonally may appear smaller. This was minimized with the interrogation of eccentric jets from a parasternal long axis view [17].

Finally, LSA-Doppler method and CMR were unable to distinguish between none/trace and mild AR in a significant number of patients with a marked overlap. Nevertheless, none of the cases with a non/trace AR had a RF > 14% by LSA-Doppler or > 7% by CMR. For the assessment of RF, an inter-observer coefficient of variation of 12% has been described for the LSA-Doppler [2] and of 6% for CMR imaging [5]. This method inherent variability in association with the presence of a small diastolic regurgitant volume due to coronary perfusion, might explain the overlap in this group of patients.

Although studies were performed on the same day in most patients, they were not performed simultaneously. Thus, differences in hemodynamic conditions might have resulted in different RF, especially in patients with mild or mild to moderate AR, where small changes in hemodynamics may influence the grading of AR. Nevertheless, a uniform tendency of overestimation with TTE derived flow convergence method makes this hypothesis less probable. Moreover, this reflects normal clinical practice during the evaluation of patients with AR, and may not be expected to have a major impact on the results, particularly in patients with moderate and severe AR.

The lack of a robust gold standard reference method makes challenge to determine with certainty which method under or overestimate. However, our study used a recommended echocardiographic multiparametric approach [10, 11] as reference standard. Data from TAVR studies using CMR for AR severity grading showed a reduced survival with a RF over 20 and 30%, supporting the use of a cutoff of > 20% as moderate AR in this group of patients. When evaluating native valvular regurgitation, in the study from Myerson et al. [18], even though a cut-off value of a RF > 33% identified patients who progressed to symptoms and surgery at a median follow-up of 2 years with a positive predictive value (PPV) of 84%, the mean RF value in the crossover group was 42 ± 9% with a PPV of 90%.

We conducted the study in a moderately sized group of patients with AR of the native valve and may not apply to those patients with prosthetic valve or atrial fibrillation. Moreover, the proposed grading scale showed a good correlation and diagnostic accuracy when compared with an accepted standard multiparametric TTE approach, but were not based on clinical outcomes. It is a hypothesis trigger showing that grading scales are not the same in all modalities and they should be adapted. Thus, future studies with an even larger number of patients will have to validate these grading scales, and appraise its correlation with clinical outcomes in different clinical scenarios.

## Conclusions

The results of the present study demonstrate that the quantitative RF values for AR grading using PISA-method, LSA-Doppler and CMR correlate well with each other but differ in groups with mild and moderate AR when using a recognized multiparametric echocardiographic approach.

## Electronic supplementary material

Below is the link to the electronic supplementary material.

Supplementary file1 (PDF 270 kb)
