# Supplementary material for: Grading of aortic regurgitation by cardiovascular magnetic resonance and pulsed Doppler of the left subclavian artery: harmonizing grading scales between imaging modalities
Source: Int J Cardiovasc Imaging. 2020 Apr 18;36(8):1517–26. doi: 10.1007/s10554-020-01844-2 (PMC7381459; doi:10.1007/s10554-020-01844-2)
Supplement: Supplementary file 1 — Supplementary file1 (PDF 270 kb) [file 10554_2020_1844_MOESM1_ESM.pdf]

Online Resource 1 Same grading scale for aortic regurgitation: Agreement of a multiparametric transthoracic echocardiography approach (TTE) with cardiovascular magnetic resonance (CMR) and Doppler of the left subclavian artery (LSA).

	TTE						
	**Mild AR**	**Moderate AR**	**Severe AR**			**S**	**M-S**
***CMR* #**							
**Mild**	**23**	5		28 (45.9%)	Sensitivity	61.5%	86.4%
**Moderate**		**7**	10	17 (27.9%)	Specificity	100%	100%
**Severe**			**16**	16 (26.2%)			
	23 (37.7%)	12 (19.7%)	26 (42.6%)	**46**/61 **(75.4%)**	Accuracy	83.6%	91.8%
***LSA Doppler* ¶**							
**Mild**	**22**	2		24 (39.3%)	Sensitivity	50%	94.7%
**Moderate**	1	**10**	13	24 (39.3%)	Specificity	100%	95.7%
**Severe**			**13**	13 (21.3%)			
	23 (37.7%)	12 (19.7%)	26 (42.6%)	**45**/61 **(73.8%)**	Accuracy	78.7%	95.1%

- Grading scale for CMR and LSA Doppler: mild, <30%; moderate, 31% to 49%; and severe, >50%

- Diagnostic test analyses: first column **S**, for severe AR; second column **M-S**, for moderate to severe AR.

- Overall agreement: # **Kappa 0.628** (p <0.0001); ¶ **Kappa 0.616** (p <0.0001).

From: Grading of aortic regurgitation by cardiovascular magnetic resonance and pulsed Doppler of the left subclavian artery: Harmonizing grading scales between imaging modalities. Ricardo A. Spampinato, Cosima Jahnke, Ingo Paetsch, Sebastian Hilbert, Susanne Löbe, Frank Lindemann, Elfriede Strotdrees, Gerhard Hindricks, Michael A. Borger. The International Journal of Cardiovascular Imaging.
